# Prevalence and determinants of polypharmacy in cardiovascular patients attending outpatient clinic in Ethiopia University Hospital

**DOI:** 10.1371/journal.pone.0234000

**Published:** 2020-06-01

**Authors:** Yonas Getaye Tefera, Mekuriaw Alemayehu, Gashaw Binega Mekonnen

**Affiliations:** 1 Department of Clinical Pharmacy, School of Pharmacy, College of Medicine and Health Sciences, University of Gondar, Gondar, Ethiopia; 2 Institute of Public Health, College of Medicine and Health Sciences, University of Gondar, Gondar, Ethiopia; Addis Ababa University School of Public Health, ETHIOPIA

## Abstract

**Background:**

While there are advances in medicine and pharmaceutical care, the burden of medication use has also grown with polypharmacy. In this regard, cardiovascular patients are subjected to polypharmacy for a longer period.

**Objective:**

The present study aimed to assess the prevalence and predictors of polypharmacy in cardiovascular outpatients attending the University of Gondar Comprehensive specialized hospital, northwest Ethiopia.

**Methods:**

A hospital-based cross-sectional study was employed at the University of Gondar Comprehensive Specialized Hospital from March 30 –May 30, 2019. The unique medical registration number of 424 patients was selected by using systematic random sampling to trace the medical chart and followed with an interview to explore the factors related to polypharmacy. All the Statistical analysis was carried out using Statistical Package for Social Sciences (SPSS) version 22. Bivariable and multivariable logistic regressions were used to identify the predictors of polypharmacy in cardiovascular patients.

**Result:**

The mean age of the respondents was 56.83 ± 15.27 years. The mean number of medications per patient was 3.3±1.6. The prevalence of polypharmacy was 24.8% in cardiovascular outpatients while cardiovascular specific polypharmacy was 9.2%. Elderly (aged ≥ 65 years and above) patients were nearly two times more likely to had polypharmacy prescriptions with AOR: 1.97; 95% CI: 1.08–3.61; p = 0.027. Patients with abnormal weight (underweight AOR: 4.51; 95% CI: 1.42–14.30; p = 0.010, overweight AOR: 3.78; 95% CI: 1.83–7.83; p<0.001 and obese AOR: 5.1; 95% CI: 2.04–12.75 p<0.001) are more likely to have polypharmacy. Having a family history of CVD increase the likelihood of polypharmacy more than double; AOR: 2.40; 95% CI: 1.17–4.93; p = 0.017. A unit increase in Charlson comorbidity index score resulted in a nearly threefold likelihood of polypharmacy with AOR: 2.83; 95% CI 1.91–3.89; p<0.001.

**Conclusion:**

One out of four cardiovascular patients attending the outpatient clinic was on polypharmacy. The elderly age, abnormal body mass index (non-normal weight), family history of cardiovascular diseases and increasing Charlson morbidity index were the predictors of polypharmacy in cardiovascular patients. Clinicians should ensure the relevance of all prescribed medications and pharmaceutical care targeting at the prevention of inappropriate polypharmacy would be pivotal to reduce polypharmacy associated burdens.

## Introduction

There is no universally agreed definition for polypharmacy, but it has been identified as the regular intake of five or more medicines in most of the literatures [1–3]. While there is advances in medicine and pharmaceutical care, the burden of medication use has also grown with polypharmacy. As of 2010, individuals using five or more prescription drugs increased by 70% as compared to the previous decade [4].

Cardiovascular diseases (CVDs) are a group of disorders which are inter-related with each other as one disorder could appear as a complication of the other. Therefore, cardiovascular patients are prone to multimorbid conditions and multiple medications which in turn exposed them for various medication-related burdens [5, 6]. Polypharmacy was known for its highest prevalence in the elderly population as consistently reported in available literature. Despite this fact, two-thirds of all polypharmacy occurred in those aged less than 70 years old [7]. This becomes more vivid in cardiovascular patients due to the complexity of the disease nature and multimorbid vulnerability which may lead to multiple medications. Therefore, polypharmacy use is not only relevant to elderly individuals but also a concern of the general cardiovascular population [3].

The worldwide prevalence estimates of polypharmacy are ranged from 9% to 39% and nowadays the prevalence has further increased, probably because of the increasing availability of new treatment options particularly in the elderly population [8, 9]. Cardiovascular disorders are the most common group of disease entities identified with the highest polypharmacy prescriptions [10] and up to 82% prevalence has been reported in elderly CVD patients [11]. Cardiovascular patients are subjected to polypharmacy for a longer duration [9, 12]. Several factors have been shown to be associated with polypharmacy in CVDs such as the development of complications, presence of various morbidities, advanced disease stage and increasing age are among others [13]. Polypharmacy in cardiovascular patients is associated with negative outcomes such as acute kidney injury, several adverse effects, and drug interaction risks [14, 15]. It increases the occurrence of drug-drug interactions and adverse drug reactions [1] and is associated with a higher risk of falls, hospitalization, poor functional status, medication non-adherence, inappropriate prescribing, morbidity and mortality [13, 16–19]. Therefore, Polypharmacy in cardiovascular patients is one of the causes of various drug-related problems and unnecessary medication burdens. These need to be prevented and managed by pharmaceutical care involving medication review and optimization of the prescribed medication.

Besides to these, there are also mounting arguments in cardiovascular polypharmacy; some suggest it should not be assumed as hazardous and represents poor care might be moderated in the context of CVDs while others recommend cardiovascular polypharmacy was underestimated and polypharmacy should no longer be based on the number of drugs but the number of pharmacologically poly active drugs in the cardiovascular system [20, 21]. Pill burden with polypharmacy imposes the heaviest medication routine in organizing and managing their daily regimens. To overcome these problems, a fixed-dose combination pill (‘polypill’) for the prevention of CVD has been recently proposed. Such polypill might be cost-effective, increase patient adherence and an affordable strategy for CVD prevention and management[13, 22, 23].

The extent of polypharmacy and patient medication experience become more important components of patient medication-related quality of life. While achieving the good therapeutic outcome and meeting the drug-related needs of patients, reduction of polypharmacy and improving the medication experiences are at the forefront of pharmaceutical care. Knowing the extent of polypharmacy in cardiovascular patients is crucial since polypharmacy is an increasing problem which has been associated with potentially inappropriate prescribing and adverse health outcomes particularly in the elderly cardiovascular patients [24]. The polypharmacy has been also highly related with increasing medication-related burden in cardiovascular patients by posing different routine management, interference with daily activities, adverse effects, drug interactions, cost and treatment-related negative psychosocial experiences [12, 13, 24–26]. Furthermore, it is important to assess polypharmacy from a clinical and public health perspective since it can be a marker of multimorbidity, inappropriate prescribing and adverse drug reactions. To the best of literature search done the prevalence of polypharmacy and its determinants were not studied exclusively at cardiovascular patients in Ethiopia. Therefore, this study aimed at assessing the prevalence and predictors of polypharmacy in cardiovascular patients attending the outpatient clinic of the University of Gondar Comprehensive Specialized Hospital in Ethiopia.

## Patients and methods

### Study setting and period

The study was conducted at the University of Gondar Comprehensive Specialized Hospital (UoGCSH) outpatient clinic from March 30 –May 30, 2019. It has both in-patient and outpatient departments. The outpatient department’s ambulatory care is given for hypertensive, heart failure, diabetic, asthmatic, epileptic, psychiatric other chronic disease patients. It is estimated that the chronic ambulatory care clinic is anticipated to serve more than 10,000 cardiovascular outpatients living in northwest Ethiopia.

### Study design and population

A hospital-based cross-sectional design was employed in cardiovascular patients who visited the ambulatory clinic of UoGCSH from March 30 –May 30, 2019.

### Sample size calculation

Single population proportion formula was used to determine the sample size.

### Study variables

#### Dependent variables

The outcome variables to be investigated were Polypharmacy andCardiovascular specific polypharmacy.

#### Independent variables

The independent variables were socio-demographic characteristics of the patient including age, gender, residence, educational status, occupation, income, social drug use and medical, clinical and medication information such as Body mass index (BMI), comorbid conditions, past medical history, number of years with CVD since diagnosis, duration of treatment, number of medical conditions, presence of CVD complications, Charlson comorbidity index score, number of medications and medication regimens.

### Data collection method

Evaluation checklist and review of medical charts to explore determinants of polypharmacy in cardiovascular outpatients were conducted by using the medical registration number of patients who were selected by systematic random sampling for interviews. Data collectors and principal investigator directly fill the questionnaire by searching the relevant information available on patients’ medical records. Data were collected by three trained nurses working in the ambulatory clinic. The weight and height of patients were measured in the ambulatory outpatient clinic by nurses working there who were data collectors of this study. Weight and height were measured with participants standing and wearing light clothing. Participants stood upright with the head in the Frankfort plane for height measurement. Bodyweight (kg) was measured using an electronic scale to the nearest 10 g, and standing height was measured using a wall stadiometer to the nearest 1 cm. Body mass index (BMI) was calculated as body weight (kg)/height (m2). The subjects were then classified into four groups according to the WHO BMI cut-offs [27] as Underweight: BMI <18.5 kg/m2; Normal weight: BMI = 18.5–24.9 kg/m2; Overweight: BMI = 25–29.9 kg/m2 and Obese: BMI ≥30 kg/m2. Self-reported health status is assessed subjectively with the patient’s perception as poor, moderate and good. The patients are also asked to respond to their physical activity habit subjectively from their daily lifestyle which is operationalized as sedentary is for those who are frequently spent by home sit, office work and with limited mobility. Moderate exercise is for those with moderate mobility work while vigorous exercise is for those spending their activity by farming, daily labor work, and extensive weight-bearing activity. We have also used the operational definitions for the following variables during data collection and interpretation of the findings. If the patient is taking five or more medications on regular basis considered as Polypharmacy (Patient-level Polypharmacy) [1–3] and cardiovascular polypharmacy is a regular intake of five or more medication which specifically active in the cardiovascular system and indicated for CVDs treatment. Primary Cardiovascular diagnosis is defined as the condition in which the patient was primarily diagnosed and being treated regularly with high attention while others considered as complication/co-morbidity and get their respective treatment. Charlson comorbidity index score is a quantitative measure of an individual’s disease burden who is living with co-morbid conditions and scored based on a sum of the number of conditions that are each assigned an integer weight from one to six [28].

### Data quality assurance

Data collectors were trained intensively on the contents of the questionnaire, data collection methods, and ethical concerns. The data collection tool was pilot tested in 20 medical charts and particpants before starting the actual data collection. The questions were translated into Amharic with modifications and back-translated to English to conform retaining its original meaning and to maintain unbiased response. The filled questionnaire was checked daily for completeness by the principal investigator.

### Data entry, analysis, and interpretation

The collected data was cleaned, entered to and analyzed using Statistical Package for Social Sciences (SPSS), version 22. In the study socio-demographic characteristics, medication and disease-related information were described using frequencies, percentage, mean and standard deviation. For categorical variables, a chi-square test was performed to assess the statistical significance of the association. The student t-test was employed to assess the association between polypharmacy and the continuous variables. Hosmer and Lemeshow test for goodness of fitness used for the model fit of each variable to use in the logistic regression model and those p-value insignificant were included in the model. Bivariable and multivariable logistic regressions were used to identify the predictors of polypharmacy in cardiovascular patients. Variables included in the adjusted multivariable regression model were those variables with a p-value of less than 0.3 in the bivariable crude ratio regression analysis. The results were adjusted for patients’ demographic and disease characteristics. Odds ratio (OR) with 95% CI were also computed along with corresponding *p*-value (*p<*0.05) as cut off points for determining statistical significance.

### Ethical considerations

Ethical approval was requested and obtained from the ethical review committee of the School of Pharmacy, University of Gondar. Written consent was also obtained from each respondent after explaining the purpose of the study. Participant’s confidentiality was guaranteed by not recording their identifiers on the questionnaire.

## Results

### Socio-demographic characteristics of patients

The mean age of the respondents was 56.8 ± 15.3 years and more than a third (36.1%) of them were above 65 years old. Two thirds (66%) of the patients were females and more than half (53.8%) were married. Nearly three fourth were urban residents, 306(72.2%). More than a quarter (27.1%) of patients had good self-reported health status. The 80(18.1%) of the patients reported a family history of CVD. (Table 1).

**Table 1:** Sociodemographic characteristics of the cardiovascular patients attending the outpatient clinic of UoGCSH, 2019 (N = 424).

Sociodemographic Variables	Frequency (%)
Sex	
Male	144(34.0)
Female	280(66.0)
Age classification	
18–64 years (Non-elderly)	271(63.9)
≥ 65 years (Elderly population)	153(36.1)
Marital status	
Single	46(10.8)
Married	228(53.8)
Divorced	41(9.7)
Separated	15(3.5)
Widowed	94(22.2)
Religion	
Orthodox Christian	352(83.0)
Muslim	72(17.0)
Educational status	
Unable to read and write	201(47.4)
Able to read and write	47(11.1)
Elementary schools	51(12.0)
High school/ secondary school	69(16.3)
College and vocational training	31(7.3)
University	25(5.9)
Occupational Status	
Student	14(3.3)
Government Employee	60(14.2)
Private Employee	11(2.6)
Merchant /private business/self-employed	35(8.3)
NGO employed	2(0.5)
Farmer	35(8.3)
Housewife	209(49.3)
Retired	46(10.8)
other	12(2.8)
Monthly Income	
Less than 35 USD	188(44.3)
35–88 USD	124(29.2)
88–176 USD	80(18.9)
Greater than 176 USD	32(7.5)
Residence	
Urban	306(72.2)
Rural	118(27.8)
Smoking History	
Yes	2(0.5)
No	422(99.5)
Alcohol Intake	
No	347(81.8)
1-2/day	72(17.0)
3-4/day	5(1.2)
Work-related activity/Physical activity self-reported	
Sedentary	68(16.0)
Moderate exercise	310(73.1)
Vigorous exercise	46(10.8)
Self-reported health status	
Poor	31(7.3)
Moderate	278(65.6)
Good	115(27.1)
Family history of Cardiovascular disease	
Yes	80(18.9)
No	344(81.1)
BMI category of CVD patients	
Underweight	36(8.5)
Normal Weight	238(56.1)
Overweight	108(25.5)
Obese	42(9.9)

### Clinical and medication characteristics of cardiovascular patients

The mean age of respondents at the diagnosis of Cardiovascular disorders were 51.03 ± 15.7 years. The current mean body mass index of the respondents was 24.3 ± 8.8 Kg/m2. The mean number of years living with CVDs and since starting medication treatment was 5.9 ± 5.6years and 5.4±6.2 years, respectively. The mean number of medications per patient was 3.3±1.6 while the cardiovascular medications per prescription were 2.6+1.6. The mean Charlson comorbidity index score of the cardiovascular patients was 2.1 ±1.4. (Table 2).

**Table 2:** The student t-test of polypharmacy and cardiovascular specific polypharmacy among different clinical characteristics of cardiovascular patients attending the outpatient clinic of UoGCSH, 2019 (N = 424).

	Total (Mean ± SD)	Polypharmacy (Mean ± SD)		Cardiovascular specific Polypharmacy (Mean ± SD)	
		Yes	No	Yes	No
Age of the patient	56.8 ±15.3	59±15*	56±15.30	61.1±14.9	56.4±15.3
Body mass index (BMI) of the patient	24.3 ± 8.8	26.9±15.8**	23.4±4.2	25.8±7.3	24.1±8.9
Age in years at diagnosis	51. ±15.7	52.9±15.9	50.4±15.6	55.8±16*	50.6±15.6
Number of years with this diagnosis	5.9±5.6	6.6±5.9	5.6±5.4	5.7±5.6	5.9±5.6
Duration since starting treatment in Years	5.4±6.2	6.4±8.1*	5.0±5.4	6.3±11.4	5.3±5.4
Number of total medical conditions	1.8±0.8	2.4±0.8**	1.6±0.7	2.4±0.8*	1.8±0.8
Charlson co-morbidity index	2.1±1.4	3.4±1.9**	1.7±0.8	2.9±0.9**	2.0±1.4
Total Number of medications per patient	3.3±1.6	5.5±0.8**	2.6±1.0	5.7±1.0**	3.1±1.4
Total number of cardiovascular medications per patient	2.6±1.3	3.9±1.3**	2.2±0.9	5.3±0.5**	2.4±1
Total number of non-cardiovascular medications per patient	0.7 ± 1	1.5±1.3**	0.4±0.7	0.4±0.7	0.7±1
Total number of self-reported over-the-counter medications per patient	0.1±0.4	0.3±0.5**	0.1±0.3	0.1±0.2	0.1±0.4

* P-value less than 0.05

** p-value less than 0.001

**Fig 1:**
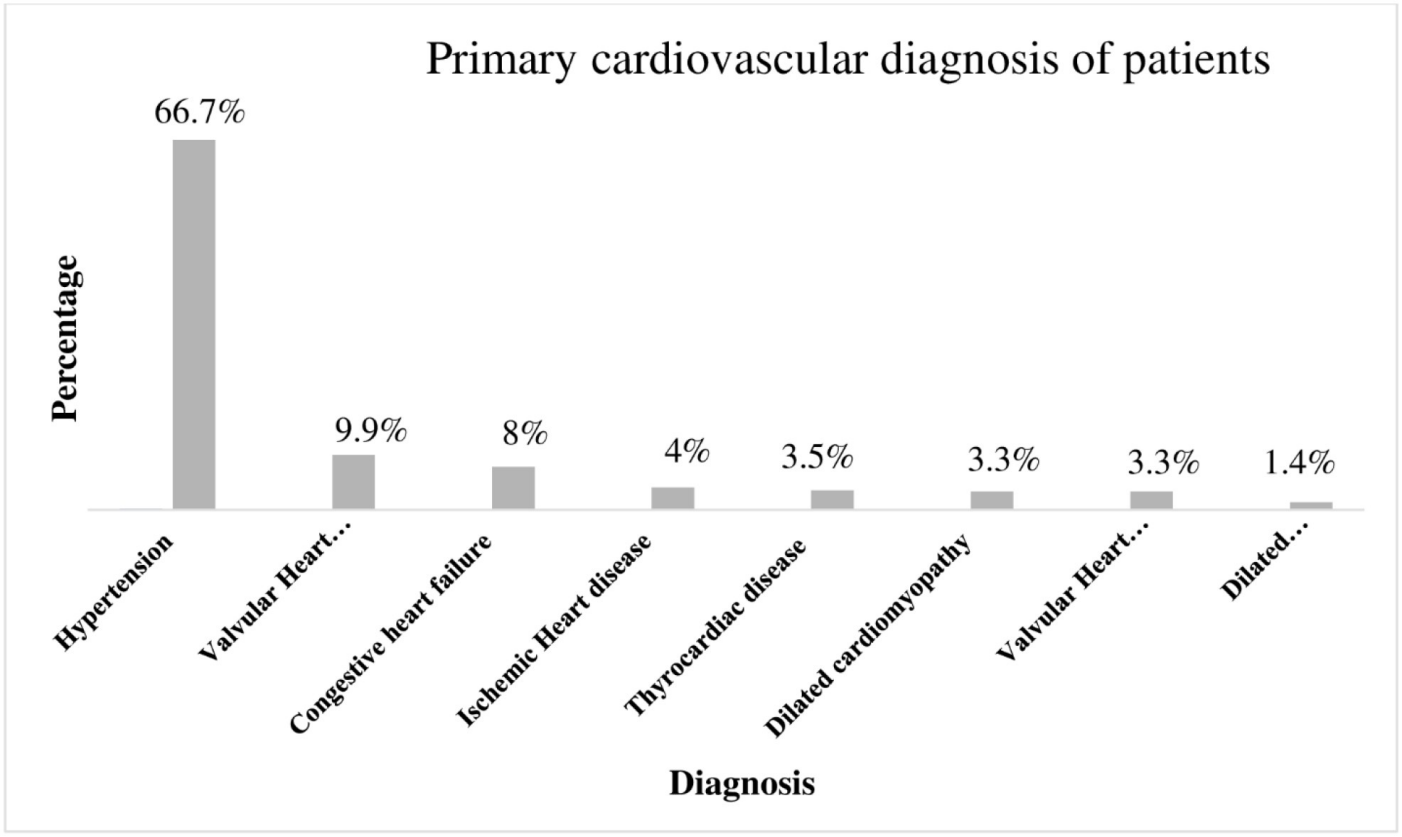
Primary diagnosis of the cardiovascular patients attending the outpatient clinic of UoGCSH, 2019 (N = 424).

**Fig 2:**
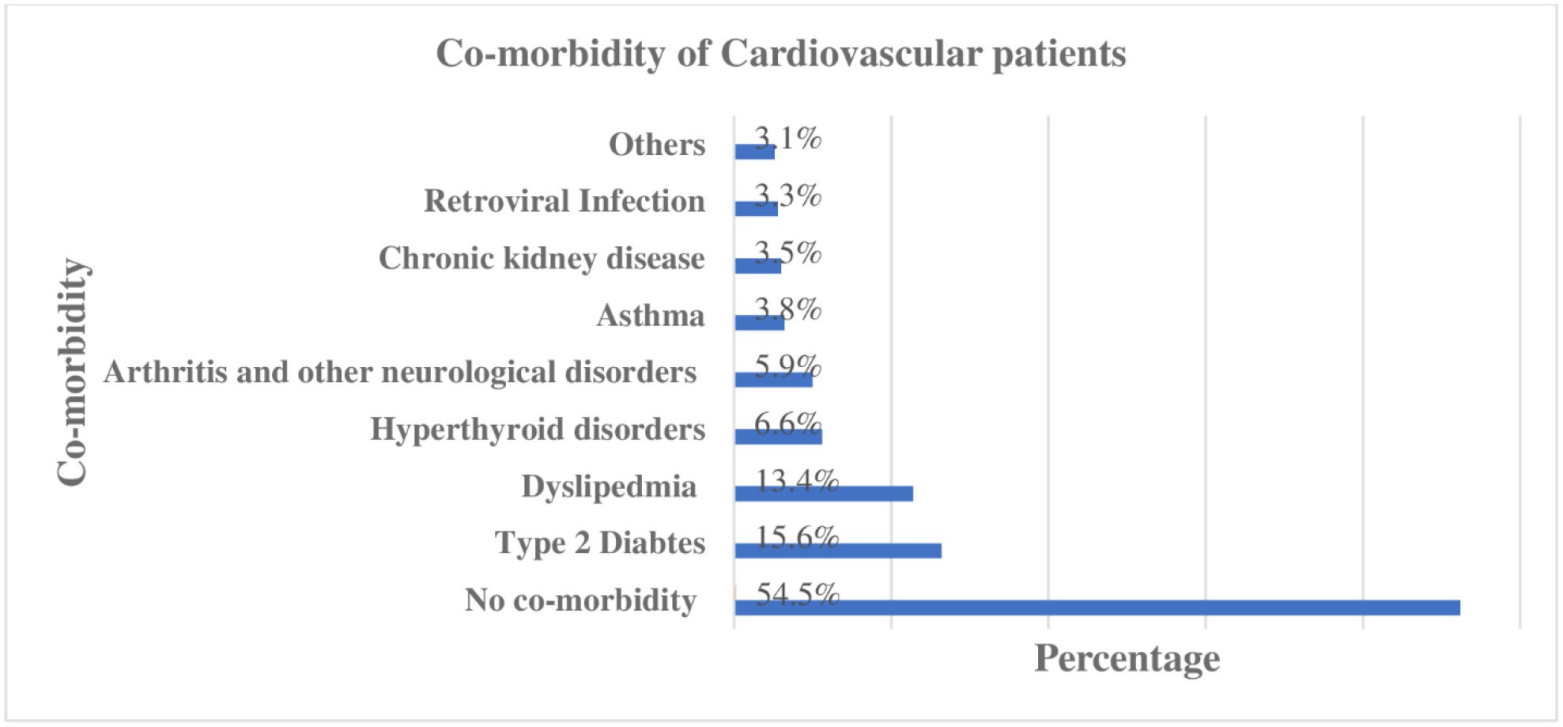
Co-morbidities in the cardiovascular patients attending the outpatient clinic of UoGCSH, 2019 (N = 424).

**Fig 3:**
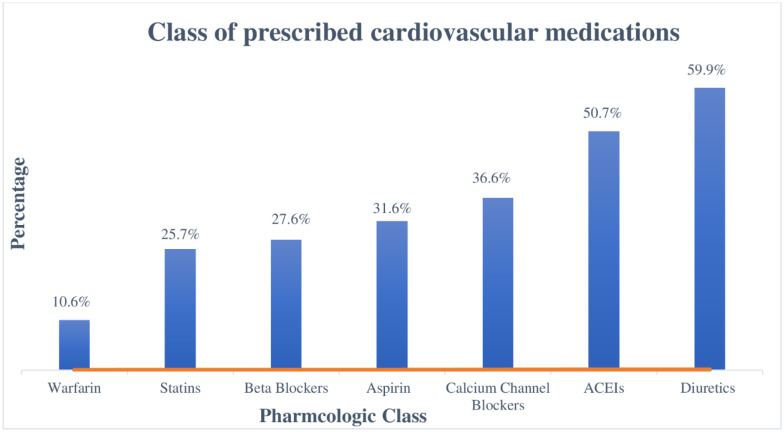
Pharmacological class of most prescribed cardiovascular medications at outpatient clinic of UoGCSH, 2019 (N = 424).

Hypertension accounted for two-thirds of the primary cardiovascular diagnosis and followed by valvular heart diseases (9.9%) (Fig 1). Type-2 Diabetes Mellitus and Dyslipidemia were the two most commonly presented co-morbidities along with CVDs in, 66(15.57% and 57(13.44%) of patients, respectively (Fig 2). A quarter (25.5%) of patients were overweight while nearly one in ten patients were either obese (9.9%) and underweight (8.5%) according to BMI category (Table 1). Diuretics (59.91%), angiotensin convertase inhibitors (50.71%) and calcium channel blockers (36.32%) were the three most prescribed cardiovascular groups of medications. (Fig 3).

### Prevalence of polypharmacy

The prevalence of polypharmacy was 24.8% in cardiovascular outpatients while cardiovascular specific polypharmacy was 9.2%. The prevalence of polypharmacy in the elderly (65 years and older) cardiovascular patients were 31.4% while cardiovascular specific polypharmacy was 13.7% in the elderly population. The prevalence of polypharmacy and cardiovascular specific polypharmacy in the non-elderly population (between 18 years and less than 65 years old) were 21% and 6.6%, respectively. Both patient level (P-value, 0.018) and cardiovascular specific polypharmacy (P-value, 0.015) were associated with elderly patients. (Table 3).

**Table 3:** Pearson’s chi-square test of polypharmacy and cardiovascular specific polypharmacy in different age groups of cardiovascular patients attending the outpatient clinic of UoGCSH, 2019 (N = 424).

		Over all prevalence	Age		P-value
			18–64 years n (%)	≥65 years n (%)	
Patient level Polypharmacy	Yes	105(24.8%)	57 (21%)	48 (31.4%)	0.018
	No	319(75.2%)	214(79%)	105(68.6%)	
Cardiovascular specific polypharmacy	Yes	39(9.2%)	18(6.6%)	21(13.7%)	0.015
	No	385(90.8%)	253(93.4%)	132(86.3%)	
Total		424	271	153	

### Factors associated with polypharmacy in cardiovascular patients

Bivariable and multivariable logistic regression test was employed to explore factors associated with polypharmacy at the patient level or specific to the cardiovascular medications. Elderly (aged ≥ 65 years and above) patients were nearly two times more likely to had polypharmacy prescriptions than patients aged 18–64 years old with AOR: 1.97; 95% CI: 1.08–3.61; p = 0.027. Patients with abnormal weight (underweight (AOR: 4.51; 95% CI: 1.42–14.30; p = 0.010, overweight AOR: 3.78; 95% CI: 1.83–7.83; p<0.001 and obese AOR: 5.1; 95% CI: 2.04–12.75 p<0.001) have three to five times more likely to polypharmacy. Having a family history of CVD will increase the likelihood of polypharmacy more than double; AOR: 2.40; 95% CI: 1.17–4.93; p = 0.017. A unit increase in Charlson comorbidity index score of a patient resulted nearly a threefold likelihood of polypharmacy, AOR: 2.83: 95% CI: 1.91–3.89; p<0.001. (Table 4).

**Table 4:** Bivariable and multivariable binary logistic regression analysis of the association of polypharmacy and its predictor variables in the cardiovascular patients attending the outpatient clinic of UoGCSH, 2019, (N = 424).

Variables	Presence of Polypharmacy		Crude odds ratio 95% CI	P-value	Adjusted Odds ratio 95% CI	P-value
	Yes	No				
Age				0.018*		0.027*
18–64 years	57	214	1		1	
≥ 65 years	48	105	1.72(1.1–2.69)		1.97(1.08–3.61)	
Residence				0.03		0.830
Urban	88	218	2.4(1.36–4.24)*		1.09(0.52–2.28	
Rural	17	101	1		1	
BMI category				<0.001		<0.001
Normal weight	38	200	1		1	
Underweight	13	23	2.98(1.39–6.38)*	0.005	4.51(1.42–14.30)*	0.010
Overweight	37	71	2.74(1.62–4.65)**	<0.001	3.78(1.83–7.83)**	<0.001
Obese	17	25	3.58(1.77–7.26)**	<0.001	5.1(2.04–12.745)*	<0.001
Physical activity				0.015		0.268
Sedentary	25	43	3.88(1.44–10.43)*	0.007	2.72(0.81–9.14)	
Moderate exercise	74	236	2.09(0.85–5.13)	0.107	1.99(0.66–6.01)	
Vigorous exercise	6	40	1		1	
Family History of CVD						
Yes	29	51	2.01(1.19–3.38)*	0.001*	2.40(1.17–4.93)*	0.017
No	76	268	1		1	
Duration in year since starting treatment (Mean *± SD*)	6.44*±8*.*07*	5.02*±5*.*35*	1.03(1.00–1.07)	0.051	1.03(0.99–1.07)	0.212
Charlson comorbidity Index score (Mean *± SD*)	3.39*±1*.*85*	1.67*±0*.*84*	3.33(2.49–4.45)**	<0.001	2.83(1.91–3.89)**	<0.001
No of medical conditions (Mean *± SD*)	2.44*±0*.*84*	1.61*±0*.*66*	4.36(3.04–6.27)**	<0.001	2.53(1.77–3.60)**	<0.001
Past medical History				0.108		
Yes	15	28	1.73(0.89–3.39)		1.57(0.63–3.93)	0.336
No	90	291	1		1	
Presence of Complication				<0.001		0.480
Yes	54	92	2.61(1.66–4.11)**		1.26(0.66–2.45)	
No	51	227	1		1	
Presence of Co-morbidity				<0.001		0.199
Yes	72	117	3.77(2.35–6.03)**		1.00(0.52–1.92)	
No	33	202	1		1	

** p <0.001,

* p<0.05

The presence of cardiovascular complication increases the cardiovascular specific polypharmacy more than three times, with AOR: 3.49; 95% CI: 1.5–8.12; p = 0.04. One increase of Charlson comorbidity score and being elderly have a slight cardiovascular specific polypharmacy increment with AOR: 1.27; 95% CI: 1.02–1.69; p = 0.035 and AOR: 1.97; 95% CI: 1.08–3.61; p = 0.039, respectively. (Table 5).

**Table 5:** Bivariable and multivariable binary logistic regression analysis of the association of cardiovascular specific polypharmacy and its predictor variables in the cardiovascular patients attending the outpatient clinic of UoGCSH, 2019 (N = 424).

Variables	Presence of CV Polypharmacy		Crude odds ratio 95% CI	P-value	Adjusted Odds ratio 95% CI	P-value
	Yes (%)	No (%)				
Age				0.017		0.039
18–64 years	18	253	1		1	
≥ 65 years	21	132	2.24(1.15–4.34)*		1.3(1.14–3.87)*	
BMI category				0.007*		0.016
Normal weight	12	226	1		1	
Underweight	6	30	3.77(1.32–10.78)*	0.013	3.8(1.03–13.98)*	0.045
Overweight	13	95	2.58(1.14–5.85)*	0.024	2.77(1.09–7.05)*	0.032
Obese	8	34	4.43(1.69–11.63)*	0.002	5.38(1.70–17.00)*	0.004
Residence				0.19		0.620
Urban	30	276	1.32(0.61–2.86)		1.26(0.51–3.07)	
Rural	9	109	1		1	
Physical activity				0.011		0.063
Sedentary	13	55	2.48(0.76–8.16)	0.135	1.73(0.46–6.54)	0.417
Moderate exercise	22	288	0.80(0.26–2.44)	0.698	0.64(0.19–2.19)	0.480
Vigorous exercise	4	42	1		1	
Family History of CVD				0.26		0.155
Yes	10	70	1.55(0.72–3.33)		1.89(0.79–4.52)	
No	29	315	1		1	
Age at diagnosis (Mean *± SD*)	55.77 *±15*.*79*	50.55 *±15*.*64*	1.02(1.00–1.05)	0.049*	1.06(0.97–1.16)	0.195
Duration in years since starting treatment (Mean *± SD*)	6.34*±11*.*5*	5.27*±5*.*37*	1.02(0.98–1.07)	0.306	1.04(0.97–1.12)	0.238
Charlson comorbidity Index score (Mean *± SD*)	2.85*±0*.*93*	2.02*±1*.*40*	1.36(1.14–1.63)*	0.001	1.27(1.02–1.69)*	0.035
No of medical conditions (Mean *± SD*)	2.38*±0*.*82*	1.76*±0*.*77*	2.35(1.61–3.44)**	<0.001	1.44(0.8–2.58)	0.221
Past medical History				0.18		0.334
Yes	4	39	1.01(0.34–3.00)		0.54(0.15–1.89)	
No	35	346	1		1	
CVD Complication				<0.001		0.004
Yes	27	119	5.03(2.46–10.27)*		3.49(1.5–8.12)*	
No	12	266	1		1	
Presence of Co-morbidity				0.218		0.285
Yes	20	169	1.35(0.7–2.6)		0.65(0.29–1.44)	
No	19	216	1		1	

## Discussion

The present study sought to determine the prevalence of polypharmacy and its predictors in patients with cardiovascular disorders. A significant proportion of cardiovascular patients were found to be on polypharmacy prescriptions according to this study. Polypharmacy specific to cardiovascular medications was also considerably high. Various factors such as elderly age, increasing morbidity, and abnormal body weight have been associated to increase the occurrence of polypharmacy in cardiovascular patients.

The body mass index in this study was abnormal in nearly half of cardiovascular patients according to the WHO classification [27]. One from four patients was overweighed and nearly one from ten cardiovascular patients was either obese or underweight. This is quite lower than the study conducted in 68 participants at Gondar town, the prevalence of overweight and obesity were 32.4% and 16.2%, respectively. The difference might be due to the small sample size used in the former study. Despite the difference, a significant increment of BMI observed from the last two decades back in which obesity was found to be 0.9% in men and 6% in women [29]. This implies overweight and obesity is also increasing in Ethiopia which needs considerable public attention as it is one of the risk factors for cardiovascular events and complications [30]. The mean Charlson comorbidity index score of cardiovascular patients (2.09±1.39) in this study is quite lower than the CCI score of a prior study conducted to assess the medication-related quality of in the elderly polypharmacy patients (*2*.*5*±*1*.*3*). But the CCI score of the polypharmacy subgroups (3.39*±1*.*853*) is higher in the current study [31]. This might be due to the later study conducted in general elderly patients while the current study of polypharmacy of cardiovascular patients who are prone and associated with multimorbidity.

The majority of patients’ primary cardiovascular diagnosis was Hypertension followed by Valvular heart disease. This finding was consistent with the previous study conducted in the same setting [32]. Type 2 diabetes mellitus and dyslipidemia were the frequent encountered compelling indications along with other CVDs. Since a relatively high prevalence of cardiovascular risk factors like dyslipidemia is common in this special population [32, 33]. Diuretics, angiotensin convertase enzymes, and calcium channel blockers were the most prescribed pharmacological class of cardiovascular drugs in the outpatient clinic. It is in agreement with the previous study conducted in the same setting [34].

The prevalence of polypharmacy in cardiovascular patients attending outpatient clinic found to be 24.8% and after precluding the non-cardiovascular medications the prevalence of cardiovascular specific polypharmacy was 9.2%. The prevalence of polypharmacy and cardiovascular specific polypharmacy in the elderly segment of the cardiovascular population aged 65 years and above were 31.4% and 13.7%, respectively. It is more than two times the polypharmacy in all outpatient prescriptions reported in 2013 by Admassie et al study in the same setting [35]. Despite the population difference studied, this could also show the escalating tide of polypharmacy overtime in Ethiopian healthcare settings likewise the rising trend elsewhere [1, 36, 37]. This is higher than Castioni et al 2017 study, the prevalence of polypharmacy in the Swedish middle age general population was 11.8% [38] and nearly equivalent to the UK polypharmacy prevalence which was 22.8% at the age of 69 [39]. The difference in the present study could be explained as cardiovascular patients were prone to polypharmacy than the general population in addition to the setting and population difference [12]. Polypharmacy of elderly cardiovascular patients in the present study is lower than the Mohammed et al 2014 study, which reported up to 84% of polypharmacy in Saudi elderly cardiac patients. Though this vast difference might be due to the stringent classification of polypharmacy (regular intake of 5 or more medicines) in the present study used while the later used 4 or more medicines as polypharmacy used to calculate the polypharmacy and included cardiac patients only treated as an inpatient [11]. While in another Korean study, the prevalence of polypharmacy in the elderly population reached 86.4% [40]. Besides the socioeconomic dissimilarities which affect patient care, healthcare facility variations and age differences; patients admitted for inpatient management also had a higher likelihood of polypharmacy than those treated as outpatients. Cardiovascular specific polypharmacy (9.2%) was reduced by more than half of the patient level polypharmacy (24.8%) by excluding all non-cardiovascular medications used for the management comorbidity. It previews the extent of multimorbidity in the cardiovascular patients was rampant and non-cardiovascular medications had a significant contribution to the patient level polypharmacy [10, 41].

Elderly age (age ≥ 65 years old) increases the likelihood of cardiovascular patient’s polypharmacy by two times in the present study. This is similar to various studies conducted elsewhere [3, 36] since polypharmacy has been related to old age and expected to rise with patients' age due to the increasing pathologies associated with aging [10, 38, 41]. A unit increase of Charlson morbidity index score and a number of medical conditions were associated with nearly threefold likely to had polypharmacy. It is similar to the primary care cross-sectional study conducted in Scotland, Polypharmacy was significantly more common in chronic heart failure patients with extreme comorbidity [42]. This would be explainable as every increase of medicine prescription was usually aimed to improve the patients worsened condition or for other clinical indications of multimorbidity. Therefore, as cardiovascular patients' multimorbid conditions increased, the likelihood of polypharmacy raises in similar fashion [37].

Abnormal body mass index’s namely underweight, overweight and obesity increase the likelihood of polypharmacy by three to five folds in cardiovascular patients. This finding is also consistent with available literature [38, 43]. Similarly in Castoni et al retrospective study in Switzerland overweight and obesity increases the likelihood of polypharmacy by two and four times, respectively [38]. The explanation would be obesity and overweight are known cardiovascular events risk factors and associated with multiple morbidities such as dyslipidemia, diabetes, and hypertension which frequently require multiple treatments [30, 38]. Underweight is the condition when the body failed to maintain normal weight which could be also a sign of multimorbidity needing multiple therapies.

In the multivariable binary logistic regression, increasing Charlson comorbidity index score, presence of cardiovascular complications and abnormal body index were the predictors to the Cardiovascular specific polypharmacy in this study. This stratification was used to understand the determinants of Cardiovascular specific polypharmacy if any, were different from the patient level of polypharmacy. More or less similar determinant factors were found with patient-level polypharmacy. Sex, residence, educational status, monthly income and self-reported health status in this study were not associated with polypharmacy after adjusting all variables in the regression model. There were conflicting results in various studies reported while low educational status, poor self-reported health, and lower-income have been related to higher polypharmacy [38, 44, 45]. This difference may be as culturally, socioeconomically and patient experience variation might be the reasons for inconsistency of the findings.

### Limitation of the study

Some of the independent variables such as self-reported health status and physical activity were assessed by the patient’s perception and have not been measured objectively by using standard tools which might make the responses to be subjective. Though it is the first study to examine the prevalence of polypharmacy and predictor variables in the cardiovascular patients at the Ethiopian healthcare setting; we did not evaluate the appropriateness of the polypharmacy in this study. Therefore, assessing the appropriateness of polypharmacy prescriptions in cardiovascular patients with treatment guideline adherence is warranted to evaluate the proper indication of each medication.

## Conclusion

One out of four cardiovascular patients attending the outpatient clinic was on polypharmacy and the cardiovascular specific polypharmacy was nearly ten percent. The elderly age, abnormal body mass index (non-normal weight), family history of CVDs and higher Charlson morbidity index were the predictors of polypharmacy in cardiovascular patients. A higher proportion of polypharmacy in cardiovascular outpatients has been observed. Therefore, clinicians should ensure the relevance of all prescribed medications and pharmaceutical care targeting at the prevention of inappropriate polypharmacy would be pivotal to reduce polypharmacy associated burdens.

## Supporting information

S1 Dataset (SPV)

S1 Data (DOCX)

## Abbreviations

ADE: Adverse Drug Event
ANOVA: Analysis of Variance
AOR: Adjusted Odds Ratio
BMI: Body Mass Index
CCI: Charlson Comorbidity Index
CI: Confidence Interval
CVD: Cardiovascular Diseases
DM: Diabetes Mellitus
NCD: Non-Communicable Diseases
OTC: Over-The-Counter
SPSS: Statistical Package for Social Sciences
UoGCSH: University of Gondar Comprehensive Specialized Hospital
WHO: World Health Organization
