# Correction: Prevalence and determinants of polypharmacy in cardiovascular patients attending outpatient clinic in Ethiopia University Hospital

**DOI:** 10.1371/journal.pone.0236328

**Published:** 2020-07-14

**Authors:** Yonas Getaye Tefera, Mekuriaw Alemayehu, Gashaw Binega Mekonnen

The affiliation for the third author is incorrect. Gashaw Binega Mekonnen is not affiliated with #2 but with #1: Department of Clinical Pharmacy, School of Pharmacy, College of Medicine and Health Sciences, University of Gondar, Gondar, Ethiopia.
