# Supplementary material for: Prevalence and determinants of polypharmacy in cardiovascular patients attending outpatient clinic in Ethiopia University Hospital
Source: PLoS One. 2020 Jun 1;15(6):e0234000. doi: 10.1371/journal.pone.0234000 (PMC7263581; doi:10.1371/journal.pone.0234000)
Supplement: S1 Data — (DOCX) [file pone.0234000.s002.docx]

# Annexes

## Annex 1: Data collection tool

***Questionnaire Number --------- Data collector name--------* Card registration number**----------

1. **Sociodemographic characteristics**
2. Age ----------------- years
3. Sex --------
4. Male
5. female
6. Weight--------------Kg
7. Height--------------Meter
8. BMI-----------
9. Marital status
10. Single
11. Married
12. Divorced
13. Separated
14. Widowed
15. Religion
16. Orthodox
17. Muslim
18. Protestant
19. Catholic
20. Others (specify)------------------
21. Educational status
22. Unable to read and write
23. Able to read and write
24. Elementary schools
25. High school/ secondary school
26. College and vocational training
27. University
28. Occupation
29. Student
30. Government employ
31. Private employed
32. Merchant /private business/self-employed
33. Farmer
34. Housewife
35. NGO employed
36. Others __________________
37. Retired
38. Monthly Income
39. Less than 1000 ETB
40. 1000-2500 ETB
41. 2500-5000ETB
42. Greater than 5000 ETB
43. Residence
44. Urban
45. Rural
46. Smoking History
47. Yes B. No
48. If Yes to Q.10 How Many cigarettes per day----------------
49. Current smoking
50. Yes B. No
51. If Yes to Q.11 How Many cigarettes per day----------------
52. Alcohol Intake
53. No
54. 1-2
55. 3-4
56. More than or equal to 5
57. -------- if different
58. Work-related activity/Physical activity
59. Sedentary
60. Moderate exercise
61. Vigorous exercise
62. Self-reported Health
63. Poor
64. Moderate
65. Good
66. **Medical and clinical information**
67. Cardiovascular diagnosis ----------
68. Do you have a family history of cardiovascular diseases? Yes------No------
69. Age at diagnosis----------------
70. Number of years with this diagnosis------?
71. comorbidity/Compelling diseases--------------
72. Duration since starting treatment--------------
73. Past medical history--------------------
74. Presence of complication-----------------------
75. Number of medical conditions ------------
76. Charlson co-morbidity index-------------
77. **Medication information**
78. Total Number of medications -------------
79. Total number of cardiovascular medications --------------
80. Total number of non-cardiovascular medications-------------
81. Total number of Over the counter medications---------------
82. Medication regimens/prescription
83. OTC medication
84. Type of dosage form ……
85. Frequency on daily basis----------

**Charlson Comorbidity Index**

| Condition | Variable name | Points | Notes |
| --- | --- | --- | --- |
| Myocardial infarction | Ml | 1 |  |
| Congestive heart failure | CHF | 1 |  |
| Peripheral vascular disease | PVC) | 1 |  |
| Cerebrovascular disease or transient ischemic disease | CVA | 1 | CVA only |
| Hemiplegia | PLEGIA | 2 | If hemiplegia, do not count CVA separated |
| Pulmonary disease/ asthma | COPD | 1 |  |
| Diabetes | DM | 1 | DM only |
| Diabetes with end-organ damage | DMENDORGAN | 2 | If end-organ damage, do not count DM separated |
| Renal disease | RENAL | 2 |  |
| Mild liver disease | MILDLIVER | 2 |  |
| Severe liver disease | SEVERELIVER | 3 |  |
| Gastric or peptic ulcer | ULCER | 1 |  |
| Cancer (lymphoma, leukemia. solid tumor | CANCER | 2 | Nonmetastatic cancer only |
| Metastatic solid tumor | METASTASES | 6 | If Metastatic, do not count cancer separated |
| Dementia or Alzheimer's | DEMENTIA | 1 |  |
| Rheumatic or connective tissue disease | RHEUMATIC | 1 |  |
| HIV or AIDS | HIV | 6 |  |
| Hypertension | HTN | 1 |  |
| Skin ulcers/ cellulitis | SKIN ULCER | 2 |  |
| Depression | DEPRESSION | 1 |  |
| Warfarin | WARFARIN | 1 |  |

የመጠይቁ ቁጥር፡ ---------- የመረጃ ሰብሳቢው ስም፡ ---------------የህክምና መዝገብ ቁጥር ---------

**ክፍል አንድ፡ ማህበረሰባዊ እና ስነ-ህዝባዊ ሁኔታ**

1. እድሜ-------------------
2. ፆታ፡ 1. ወንድ 2. ሴት
3. ክብደት-------በኪ.ግ
4. ቁመት--------በሜትር
5. ቢማይ --------
6. የጋብቻ ሁኔታ፡
7. ያላገባ/ች 2. ያገባ/ች 3. የተፋታ/ች 4. የተለያየ/ች 5. በሞት ያጣ/ች
8. ሐይማኖት፡ 1. ኦርቶዶክስ ክርስትና 2. ኢስላም 3.ፕሮቴስታንቲዝም 4.ካቶሊክ 5.ሌላ (ይገለፅ)-
9. የትምህርት ደረጃ (ያጠናቀቁት ወይም በመማር ላይ ያሉት)፡

1.ማንበብና መፃፍ የማይችል/ትችል 2. ማንበብና መፃፍ ብቻ የሚችል/ምትችል

3.የመጀመሪያ ደረጃ ት/ት (1-8) 4. የሁለተኛ ደረጃ ት/ት (9-12)

5.ኮሌጅ/ቴክኒክና ሙያ 6. የዩኒቨርሲቲ ት/ት

1. ዋና ስራ፡

1.ተማሪ 2. የመንግስት መ/ቤት ሰራተኛ 3.የግል ድርጅት ሰራተኛ 4.የግል ስራ(ቢዝነስ) 5.የእርዳታ ድርጅት (ኤንጂኦ) 6.ግብርና 7.የቤት እመቤት 8.ጡረተኛ 9.ሌላ (ይገለፅ)___

1. ወርሃዊ ገቢ
2. ከ1000 ብር በታች 2. ከ1000 -2500 ብር

3. ከ2500-5000 ብር 4. ከ5000 ብር በላይ

1. ቋሚ የመኖሪያ አድራሻ ፡ 1. ከተማ 2. ገጠር
2. ሲጋራ አጭሰው ያውቃሉ 1. አዎ 2. የለም
3. ለጥያቄ ቁጥር 113 መልስዎ አዎ ከሆነ በቀን ምን ያክል ሲጋራ ያጨሱ ነበር ----------
4. አሁንስ ሲጋራ ያጨሳሉ 1. አዎ 2. የለም
5. ለጥያቄ ቁጥር 115 መልስዎ አዎ ከሆነ በቀን ምን ያክል ሲጋራ ያጨሳሉ፡፡-----------
6. አልኮል በቀን ምን ያህል ይጠጣሉ

1.በፍጹም አልጠጣም

2. ከ1-2

3. ከ3-4

4. 5 እና ከዛ በላይ

5. የተለየ ከሆነ ይገለጽ --------

1. በስራ ምክንያት/በፍላጎት የሚሰራ የአካል እንቅስቃሴ ካለ ምን ይመስላል
2. የቢሮ ስራ/መቀመጥ የሚበዛው ስራ
3. መጠነኛ የሆነ የአካል እንቅስቃሴ
4. ከባድ ስራ/የአካል እንቅስቃሴ
5. በራስዎ እይታ የጤናዎ የመሻሻል ሁኔታ ምን ይመስላል

1.ዝቅተኛ

2. መካከለ|ኛ

3. ጥሩ

**ክፍል ሁለት፡ የሜድካልና ክሊኒካል መረጃ**

1. የህመሙ ምርመራ-------------------------------------------
2. ከቤተሰብ ውስጥ ከልብና ደም ዝውውር ጋር የሚያያዙ በሽታዎች ያለበት ሰው አለ

1. አዎ 2. የለም

1. ለመጀመርያ ጊዜ ህመሙ እንዳለበዎ ሲመረመሩ እድሜዎ ስንት ነበር----------------
2. ምን ያህል ጊዜ ከዚህ ህመም ጋር ቆዩ---------
3. ተጨማሪ በሽታ ካለብዎ----------------
4. ህክምናውን ከጀመሩ ምን ያህል ጊዜ ሆነ--------------
5. ያለፈ የህመም ታሪክ ካለ ቢነግሩን----------------------------
6. ከበሽታዎ ጋር ተያይዞ የመጣ የከፋ ጉዳት/በሽታ ደረጃ አለ----------------
7. ከዚህ ውጭ ምን ያህል በሽታ ታማሚ ነዎት---------
8. Charlson co-morbidity index-------------

**ክፍል ሶስት፡ ስለሚወስዱት መድሃኒቶች መረጃ**

1. በጠቅላላው ስንት መድሃኒት ይወስዳሉ -------------
2. በጠቅላላው ለልብና ደም ዝውውር ጋር ለሚያያዙ በሽታዎች የታዘዙ ስንት መድሃኒት ይወስዳሉ ---------
3. በጠቅላላው ስንት መድሃኒት በተራ ቁጥር 302 ከተጠቀሰው በሽታወች ውጭ ይወስዳሉ-------------
4. ያለሃኪም ትዛዝ የሚወስዱት መድሃኒት ቤት ካለ ቢነገሩን---------------
5. የታዘዙ መድሃኒቶች ዝርዘር
6. ያለሃኪም ትዛዝ የሚወስዱት መድሃኒት ቤት ካለ ቢነገሩን
7. የሚወስዱት መድሀኒት የተዘጋጀበት መልክ፡ ሀ)በኪኒን ለ) በካፕሱል ሐ)በመርፌ መ)በሽሮፕ
8. በቀን ስንት ጊዜ መድሃኒትዎን ይወስዳሉ----------
